# Employer and Servant

**Published:** 1907-07-13

**Authors:** 


### EMPLOYER AND SERVANT.

### THE FORMER'S LIABILITY AND RISKS.

In most of the policies which the Insurance Companies are issuing to cover workmen's compensation risks now brought by the new Act so prominently to the notice of all employers, it will be observed that the assured is indemnified against all liability under, or by virtue of, the Common Law; Employers' Liability Act, 1880; Workmen's Compensation Act, 1906; Lord Campbell's Act, 1846; in respect of all injuries which may happen to any servant while in his employ. The liability against which protection is thus given varies under the different Acts, and it will be useful at the present time to recapitulate what this liability may be.

### The Common Law Rule.

1. The Common Law does not give a servant the same right of action against an employer in respect of injuries suffered by him in the course of his employment, as it does to outside persons who happen to be injured by reason of the negligence either of the employer or anyone employed by him. The servant could only sue his employer where the employer had been himself negligent. Negligence on the part of a fellow-servant resulting in injury was regarded as one of the risks incidental to the servant's employment, and no action against the master lay in consequence of it. This was the doctrine of "common employment" so called. The Common Law rule as to the liability of the master for his own personal negligence resulting in accident to his servant still exists, and has not been affected by recent legislation, and it is important to remember this, because there is no limit to the damages which servants can recover at Common Law, as there is in cases under the Workmen's Compensation Act.

### The Employers' Liability Act.

2. The Employers' Liability Act, 1880, struck at the doctrine of common employment and largely increased the liability of the employer, and is still unrepealed. It only affects certain causes of injury and certain classes of workmen. These last can claim compensation for injuries caused by the negligence of another servant who is in a position of authority over them, or by obedience to orders and rules promulgated by the employer or by someone else with his authority.

### The Workmen's Compensation Act.

3. The Workmen's Compensation Acts, 1897 and 1900, are now repealed as from July 1 last, when their place was taken by the Act which was passed last year. The provisions of this Act are (or ought to be) matters of common knowledge, and it need only be said that the employer's liability is still further increased by making him liable to pay compensation whether he or any of his servants are negligent or not, while the classes of workmen who are entitled to its benefits have been so enlarged as to include almost everyone who works under a contract of service with a salary under £250 a year, and in the case of manual labourers, with no such limit at all.

### The Fatal Accidents Act.

4. Lastly, Lord Campbell's Act, 1846, or, to call it by its more correct name, the Fatal Accidents Act, needs a word of explanation. The Common Law rule was that the death of a person injured by the negligence of another put an end to his cause of action, which did not survive to his executors or representatives. Very great hardship resulted, because widows and children were often left without any compensation for the death of a bread-winner in circumstances which would have entitled the bread-winner himself to compensation, had he survived. Lord Campbell's Act gives a right of action to the personal representatives of anyone whose death has been brought about by the negligence of another, if the deceased would have been entitled himself to bring an action for negligence, if he had lived. This right is to be exercised for the benefit of husband, wife, parents, and children only, and is limited to the amount of the pecuniary loss suffered by reason of the deceased's death, calculated on a strictly practical (and not sentimental) basis. In many cases it will probably be more advantageous to make a claim under the new Compensation Act, with its limit of £300, but Lord Campbell's Act still remains as a concurrent remedy, though only applicable in the comparatively small number of cases where negligence can be proved.

### The New Act.

It will be seen, therefore, that the liability of the employer to his servants is not wholly governed by the new Act, but that his liability in all cases where personal negligence can be brought home to him or to those in authority under him, is considerably wider. The policies, however, which the companies are now prepared to issue fully protect him against all legal liability, and it will be found that in most cases for a small additional premium they will cover him against certain moral obligations—*e.g.*, medical expenses and the like, for which he would not otherwise have to pay. The absolute necessity for insurance on the part of every employer of servants of any kind is more than ever to be insisted upon, and much pain and suffering will be caused not only to the servant, but to the employer also, if this counsel is disregarded until it is too late.

### PRACTICAL POINTS.

### EMPLOYERS' LIABILITY INSURANCE.

The clerk to a provincial hospital writes :—

My committee will be much obliged if you will inform me whether London hospitals are making any special features of their insurances other than in respect of the ordinary paid staff and of claims therefrom under :—

The Workmen's Compensation Acts, 1897, 1900, and 1906.

Employers' Liability Act, 1880.

Fatal Accidents Act, 1846.

Common Law,

